# Impact of CYP3A4 functional variability on ziprasidone metabolism

**DOI:** 10.3389/fphar.2025.1585040

**Published:** 2025-04-29

**Authors:** Qi Zhou, Yameng Wu, Zhize Ye, Zheyan Zhang, Kai Zheng, Jianchang Qian, Zhongxiang Xiao, Yang Lu

**Affiliations:** ^1^ Affiliated Yueqing Hospital, Wenzhou Medical University, Wenzhou, Zhejiang, China; ^2^ School of Pharmaceutical Sciences, Institute of Molecular Toxicology and Pharmacology, Wenzhou Medical University, Wenzhou, Zhejiang, China; ^3^ Department of Pharmacy, Shaoxing People’s Hospital, Shaoxing, China

**Keywords:** ziprasidone, quercetin, drug-drug interaction, CYP3A4, LC-MS/MS

## Abstract

**Introduction:**

Ziprasidone is primarily metabolized by CYP3A4, an enzyme with genetic variability and susceptibility to inhibition or induction. This study explored the functional variability of CYP3A4 in ziprasidone metabolism, focusing on drug interactions and genetic polymorphisms.

**Methods:**

The metabolic inhibition and kinetic properties of ziprasidone were evaluated through in vitro experiments utilizing rat liver microsomes (RLM), human liver microsomes (HLM), and CYP3A4 baculosomes. In vivo validation studies were conducted in Sprague-Dawley rats.

**Results:**

Quercetin significantly inhibited ziprasidone metabolism in vitro, with in vivo coadministration led to marked increasing in ziprasidone’s AUC, CLz/F, and Cmax. Inhibition followed mixed mechanisms in RLM, HLM, and CYP3A4.1 systems. Analysis of CYP3A4 variants revealed distinct metabolic efficiencies: CYP3A4.3, 15, and 33 exhibited elevated clearance, while CYP3A4.24, 31, and 34 showed reduced activity. Quercetin’s inhibitory potency varied across alleles, with IC50 values of 17.59 ± 1.01 μM in CYP3A4.1 and 54.51 ± 1.35 μM in CYP3A4.33. Molecular docking identified ARG106, PHE108, PHE215, THR224, and GLU374 as key residues mediating inhibition.

**Discussion:**

The findings of this study underscore the critical role of quercetin-mediated CYP3A4 inhibition and CYP3A4 genetic polymorphisms in modulating ziprasidone metabolism.

## 1 Introduction

Ziprasidone is an atypical antipsychotic that is also associated with some side effects such as headache, insomnia, nausea, drowsiness, and respiratory disorders during treatment. Rare side effects include indigestion, weight gain, and extrapyramidal symptoms (Goodnick, 2001; Greenberg and Citrome, 2007). Seriously, it can even cause liver damage and excessive poisoning (2012). From a pharmacokinetic point of view, the increase in blood concentration in individuals is the main cause of side effects (Mauri et al., 2018). Therefore, identifying potential influencing factors can provide a reference for individualized clinical application.

Ziprasidone undergoes extensive metabolism in humans, with less than 5% of the administered dose excreted unchanged (Beedham et al., 2003). Ziprasidone metabolism is mediated by two distinct enzyme systems: (1) the cytosolic aldehyde oxidase system, which catalyzes the primary reductive pathway, and (2) cytochrome P450 3A4 (CYP3A4), responsible for two oxidative metabolic routes (Spina and de Leon, 2007; Urichuk et al., 2008). Notably, CYP3A4-mediated oxidation generates ziprasidone sulfoxide as a major metabolite (Caccia, 2000). Given that CYP3A4 activity is highly susceptible to both inhibition and induction by concomitant medications, polypharmacy in schizophrenia treatment poses significant pharmacokinetic challenges (Zhou, 2008). Frequent CYP3A4-mediated drug interactions may lead to either decreased or increased plasma concentrations of antipsychotics, potentially compromising therapeutic efficacy or elevating the risk of adverse effects. Previous studies indicate that certain antiepileptic drugs (e.g., carbamazepine, phenobarbital) can significantly reduce plasma concentrations of second-generation antipsychotics (Kennedy et al., 2013). Conversely, nimodipine coadministration increases blonanserin exposure, elevating its AUC and C_max_ by more than 2-fold (Ye et al., 2024). However, drug interaction data for ziprasidone remain limited, necessitating further investigation given its widespread clinical use. CYP3A4 exhibits substantial genetic polymorphism, leading to marked interindividual variability in metabolic activity (Werk and Cascorbi, 2014; Hu et al., 2022). To date, 48 CYP3A4 alleles have been identified globally, with CYP3A4*4, *5*, *18* and *23* being the predominant mutations in the Han Chinese population (Hu et al., 2017). According to the current report, the CYP3A4*18 mutation is the highest incidence of coding region mutations in the Han population. Functional studies reveal that CYP3A4.18 significantly enhances the intrinsic clearance of amiodarone (Yang et al., 2019) and lidocaine (Fang et al., 2017) while reducing that of regorafenib (Li et al., 2019) compared to wild-type CYP3A4.1. However, its impact on loperamide (Lin et al., 2019) and ibrutinib (Xu et al., 2018) metabolism appears negligible. These findings indicated that CYP3A4 polymorphisms may substantially influence ziprasidone’s systemic exposure, warranting further pharmacogenetic evaluation.

In this study, we employed an *in vitro* liver microsomal incubation system to investigate potential drug-drug interactions involving ziprasidone, with subsequent *in vivo* validation in a rat model. The inhibitory mechanisms were further elucidated through detailed analysis of enzyme-catalyzed reaction kinetics. Additionally, we systematically characterized the metabolic profiles of ziprasidone across CYP3A4 wild-type and 19 variants, evaluating both the impact of genetic polymorphisms on drug metabolism and the differential effects of pharmacological inhibitors. Molecular docking simulations were performed to identify key structural interactions at the molecular level. These findings provide important insights into potential clinically relevant drug interactions with ziprasidone, while also establishing a scientific foundation for precision medicine approaches that incorporate individual CYP3A4 genetic polymorphisms.

## 2 Materials and methods

### 2.1 Chemicals and reagents

Ziprasidone and its sulfoxide metabolite were purchased from Shanghai Canspec Scientific Instruments Co., Ltd. (Shanghai, China). Midazolam (internal standard, IS) was obtained from Jiangsu Nhwa Pharmaceutical Co., Ltd. (Xuzhou, China). A screening library of 50 compounds, including quercetin and resveratrol (Supplementary Table S1), was acquired from Shanghai Canspec. Nicotinamide adenine dinucleotide phosphate (NADPH) was supplied by TargetMol (Boston, MA, United States). HPLC-grade methanol, acetonitrile (ACN), and formic acid were procured from Wenzhou Jinshan Chemical Reagent Instrument Co. (Wenzhou, China). Rat liver microsomes (RLMs) and human liver microsomes (HLMs) were purchased from Corning Life Sciences (New York, NY, United States). Recombinant CYP3A4 and cytochrome b5 were prepared as previously described (Fang et al., 2017). The genetic characteristics of CYP3A4 variants are detailed in Supplementary Table S2.

### 2.2 Liquid chromatography-tandem mass spectrometry conditions

A validated LC-MS/MS method was developed for the simultaneous quantification of ziprasidone and its sulfoxide metabolite. Chromatographic separation was achieved on a BEH C18 column (2.1 × 100 mm, 1.7 μm; Waters Corp., United States) maintained at 40°C. The mobile phase consisted of (A) 0.1% formic acid in water and (B) methanol, delivered at 0.40 mL/min under the following gradient program: 0–2.5 min: 10% → 90% B; 2.5–2.8 min: 90% → 10% B; 2.8–4.5 min: 10% B (re-equilibration). Mass spectrometric detection was performed using electrospray ionization in positive mode (ESI+) with the following parameters: Ion source 4.5 kV capillary voltage, 400°C heating block; Gas flows 3 L/min nebulizing gas, 5 L/min drying gas. MRM transitions: Midazolam (IS) m/z 326.1 → 291; Ziprasidone m/z 413.2 → 194; Ziprasidone sulfoxide m/z 428.9 → 99. All analytes were baseline-resolved, with retention times of 1.99 min (midazolam), 1.94 min (ziprasidone), and 1.69 min (ziprasidone sulfoxide) (Supplementary Figure S1). No interfering peaks were observed in the chromatograms.

### 2.3 Enzymatic kinetics of ziprasidone metabolism

The incubation mixture, prepared in phosphate-buffered saline (PBS, pH 7.4), contained either 0.3 mg/mL rat liver microsomes (RLM) with ziprasidone (0.5–40 μM) or 0.2 mg/mL human liver microsomes (HLM) with ziprasidone (0.1–20 μM). After 5 min preincubation at 37°C, reactions were initiated with 1 mM NADPH and terminated after 20 min by adding 400 μL ice-cold acetonitrile, followed by spiking with 500 ng/mL IS. The samples were vortexed (2 min), centrifuged (10,075 × g, 10 min, 4°C), and the supernatant was analyzed via LC-MS/MS using calibration standards (0.01–10 μM). Metabolite formation rates were plotted against substrate concentrations to derive Michaelis-Menten kinetics (nonlinear regression) for K_m_ determination. For inhibition studies, reactions containing 100 μM inhibitor, 0.3 mg/mL RLM, and ziprasidone (10 μM, ≈ K_m_) in PBS were initiated with 1 mM NADPH, incubated for 20 min, and processed identically for LC-MS/MS analysis.

The incubation mixture was set up in phosphate-buffered saline (PBS) with 0.5 pmol of CYP3A4.1 or its variant, 2.5 μg of cytochrome b5, and 0.1–25 μM of ziprasidone, and then incubated for 20 min. Following the incubation, 400 μL of ACN and 20 μL of IS were added to the mixture. The sample was then vortexed and centrifuged, and the resulting supernatant was collected for analysis via LC-MS/MS.

### 2.4 Animal experiments

To minimize potential confounding effects of hormonal fluctuations, this study exclusively utilized male Sprague-Dawley (SD) rats (340 ± 15 g) obtained from Vital River Laboratories (Beijing, China). All experimental procedures were conducted in strict compliance with institutional guidelines and were approved by the Laboratory Animal Ethics Committee of Wenzhou Medical University (Ethical Approval No.: wydw2023-0461), following the National Research Council’s guidelines for the care and use of laboratory animals.

Following a 7-day acclimatization period under controlled conditions (temperature: 20°C–25°C; humidity: 50%–65%; 12h/12 h light/dark cycle), 18 rats were fasted for 12 h (water *ad libitum*) and randomly divided into three treatment groups (n = 6 per group). Based on clinical dose equivalency calculations, Group A received 50 mg/kg resveratrol, Group B received 50 mg/kg quercetin (both suspended in corn oil via intragastric administration), while Group C served as the corn oil vehicle control. Thirty minutes post-pretreatment, all animals received 15 mg/kg ziprasidone orally.

Serial blood samples (50 μL) were collected at predetermined time points (0, 1, 2, 4, 6, 8, 10, 12, 24, and 48 h) and processed with 150 μL acetonitrile and 20 μL midazolam IS (500 ng/mL). After vortex-mixing (2 min) and centrifugation (10,075 × g, 10 min), the supernatant was subjected to LC-MS/MS analysis. For standard curve preparation, blank plasma (40 μL) was spiked with ziprasidone and ziprasidone sulfoxide (5 μL each), followed by addition of 150 μL ACN and 20 μL IS. Calibration curves were established over 1–1,000 ng/mL for both analytes, with samples processed identically to study specimens.

### 2.5 Kinetics inhibitory assay

The microsomal and recombinant CYP3A4 incubation systems were established to evaluate quercetin’s inhibitory effects on ziprasidone metabolism. For microsomal studies, reactions were conducted in PBS containing either 0.3 mg/mL RLM or 0.2 mg/mL HLM, supplemented with quercetin (0–100 μM), ziprasidone (10 μM, approximating K_m_), and NADPH. IC_50_ determination used quercetin concentrations from 0 to 100 μM with fixed ziprasidone (10 μM), while inhibition kinetics were assessed using ziprasidone (2.5–20 μM, spanning K_m_) with quercetin at 0, 6.5, 13, and 26 μM (RLM) or 0, 6.25, 12.5, and 25 μM (HLM).

For recombinant CYP3A4 studies, the system contained 0.5 pmol CYP3A4.1 or CYP3A4.33, 2.5 μg cytochrome b5, ziprasidone (5 μM for CYP3A4.1 or 3 μM for CYP3A4.33, ≈ K_m_), quercetin (0–100 μM), and NADPH. Kinetic inhibition studies employed ziprasidone (1.25–10 μM, CYP3A4.1) with quercetin (0, 10, 20, 40 μM, ≈ IC_50_). All samples were processed and analyzed by LC-MS/MS.

### 2.6 Molecular docking

SDF format files for ziprasidone and quercetin were obtained from the PubChem database, while the CYP3A4 protein structure was sourced from the PDB database. Pymol software was employed to optimize the protein structure by removing water molecules and small molecule ligands. Hydrogenation and charge processing were carried out using AutoDock Tools, and the files were saved in pdbqt format. Molecular docking was conducted using Vina software within the PyrX platform, where binding energy calculations and output result files were generated. Finally, PyMol software was used to visualize and analyze the docking results.

### 2.7 Statistical analysis

The Lineweaver-Burk double reciprocal plot was created with GraphPad Prism 8.0. Essential pharmacokinetic parameters were determined using a non-compartmental model through Drug and Statistics (DAS) software version 3.0. For the polymorphism experiment results, one-way ANOVA was performed in GraphPad Prism 8.0, while pharmacokinetic data were assessed using an unpaired t-test, with a *p*-value below 0.05 deemed statistically significant.

## 3 Result

### 3.1 Clarify the drug interaction spectrum of ziprasidone

**FIGURE 1:**
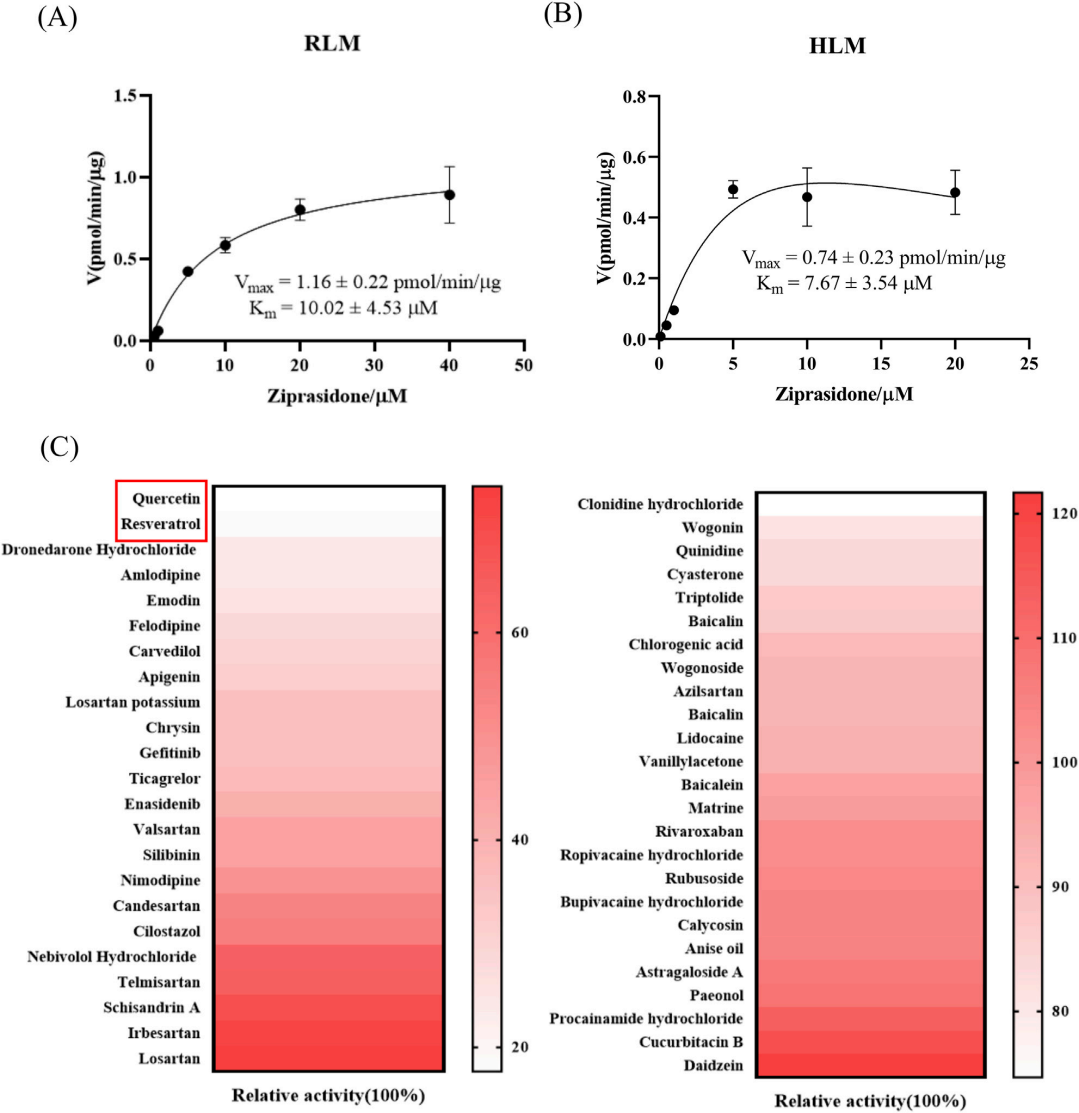
Pharmacokinetic characteristics of ziprasidone were assessed in rat liver microsomes (RLM) and human liver microsomes (HLM), along with the results of inhibitor screening. **(A)** Michaelis-Menten plots illustrating ziprasidone metabolism in RLM (n = 3). **(B)** Michaelis-Menten plots depicting ziprasidone metabolism in HLM (n = 3). **(C)** Thermogram depicting the inhibitory impact of different medications on ziprasidone metabolism.

The samples were evaluated by LC-MS/MS for the quantification of the metabolite, ziprasidone sulfoxide. The reaction rate of ziprasidone in each system was calculated. The reaction rate was used as the ordinate and the concentration of ziprasidone was used as the abscissa. The corresponding Michaelis-Menten curve and enzyme kinetic parameters of each incubation system were nonlinearly fitted by the Michealis-Menten model of GraphPad Prism 8.0 software. The Michaelis-Menten curves of each system are shown in Figure 1A,B. The findings indicated that in RLM incubation system, the K_m_ for ziprasidone was measured at 10.02 ± 4.53 μM, with a V_max_ of 1.16 ± 0.22 pmol/min/μg. Meanwhile, in HLM incubation system, the K_m_ was 7.67 ± 3.54 μM, and the V_max_ was 0.74 ± 0.23 pmol/min/μg.

On this basis, we selected drugs that can inhibit the metabolism of ziprasidone. Figure 1C and Supplementary Table S1 shows the inhibitory effect of various cardiovascular drugs and natural compounds on the metabolism of ziprasidone. The findings indicated that both quercetin and resveratrol markedly suppressed the metabolism of ziprasidone by over 80%. Therefore, these two inhibitors were selected as inhibitors to further investigate the inhibition of ziprasidone metabolism.

### 3.2 Quercetin in combination with ziprasidone altered the pharmacokinetic properties of ziprasidone in rats

**FIGURE 2:**
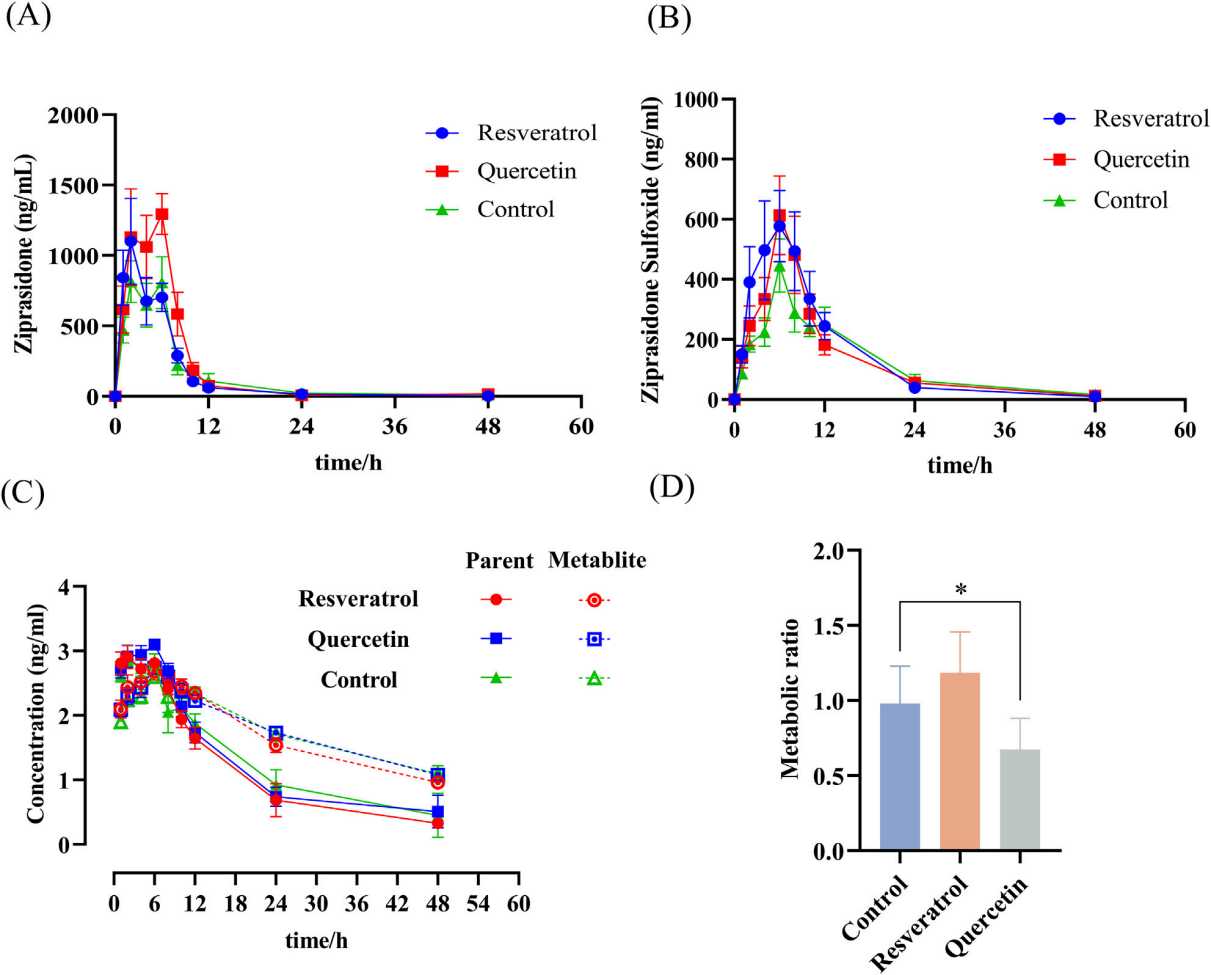
Rat pharmacokinetic study of ziprasidone and its metabolite. Prior to the study, rats underwent a fasting protocol, then received the drug, had blood drawn, and samples were processed and analyzed as detailed in the “Animal Experiments” section. A graph was created with the time of blood collection on the x-axis and the drug concentration in the blood on the y-axis. Curves for ziprasidone **(A)** and its metabolite, ziprasidone sulfoxide **(B)**, following co-administration with resveratrol or quercetin. **(C)** Semi-logarithmic concentration-time profile for ziprasidone and its metabolite based on **(A, B)**. **(D)** Ratio of drug metabolism in the bloodstream. Data are presented as mean ± SEM, n = 6.

Subsequently, we conducted an *in vivo* analysis of drug interactions. We chose to work exclusively with male rats to investigate the metabolism of ziprasidone, thereby eliminating hormonal influences on the physiological cycles of female rats. To exclude the influence of food, the rats were fasted before the experiment. The average blood concentration-time curve of ziprasidone and its metabolite ziprasidone sulfoxide for both the experimental and control groups are depicted in Figures 2A,B, respectively, with the detailed pharmacokinetic parameters presented in Tables 1,2. Compared with the control group, after oral administration of quercetin and ziprasidone, the AUC_(0-t)_ and AUC_(0-∞)_ of ziprasidone in the experimental group increased by about 142% and 134%, respectively, and C_max_ increased by about 168%. While coadministration of resveratrol and quercetin significantly increased the blood clearance (CL_z/F_) in rats by approximately 342% and 215%, respectively, compared to the control group. There was no significant difference in other pharmacokinetic parameters. Following coadministration with resveratrol or quercetin, the CL_z/F_ of ziprasidone sulfoxide increased significantly by approximately 299% and 324%, respectively, compared to the control group. No other pharmacokinetic parameters exhibited statistically significant alterations. Based on the semi-logarithmic concentration-time profile shown in Figure 2C, it was observed that the terminal phase of ziprasidone sulfoxide, following co-administration with quercetin or resveratrol, ran parallel to the initial phase. This suggests that the kinetics of the metabolites are constrained by the elimination rate. It can also be seen from Figure 2D that the plasma metabolism ratio of ziprasidone was significantly decreased in the experimental group taking both quercetin and ziprasidone. The pharmacokinetic results showed that resveratrol had no significant inhibitory effect on the metabolism of ziprasidone in rats. In conclusion, our subsequent inquiries into the mechanism and nature of inhibition primarily concentrated on the impact of quercetin on ziprasidone metabolism.

**TABLE 1:** Pharmacokinetic parameters of ziprasidone in three groups.

Group Parameters	Unit	Control	Resveratrol + Ziprasidone	Quercetin + Ziprasidone
AUC_(0-t)_	μg/L×h	6,611.13 ± 2,585.97	6,755.19 ± 2,974.98	9,386.29 ± 2,497.84*
AUC_(0-∞)_	μg/L×h	6,983.30 ± 3,075.86	6,759.08 ± 2,972.86	9,388.70 ± 2,495.97*
t_1/2z_	h	8.64 ± 10.80	4.06 ± 1.52	3.79 ± 2.11
T_max_	h	4.00 ± 2.19	4.33 ± 2.66	4.00 ± 1.79
C_max_	μg/L	962.83 ± 367.29	1,163.45 ± 672.81	1,616.56 ± 489.08*
V_z/F_	L/kg	24.33 ± 19.05	56.15 ± 42.71	33.34 ± 22.14
CL_z/F_	L/h/kg	2.63 ± 1.37	9.00 ± 4.54*	5.65 ± 1.48*

Note: Compared to control group, *P < 0.05. The results are expressed as mean ± standard deviation (SD).

**TABLE 2:** Pharmacokinetic parameters of ziprasidone sulfoxide in three groups.

Group Parameters	Unit	Control	Resveratrol + Ziprasidone	Quercetin + Ziprasidone
AUC_(0-t)_	μg/L×h	5,831.98 ± 1,744.78	7,562.09 ± 3,861.09	6,228.98 ± 2,876.72
AUC_(0-∞)_	μg/L×h	6,101.07 ± 1,921.68	7,629.29 ± 3,819.91	6,490.29 ± 2,653.93
t_1/2z_	h	9.34 ± 3.46	6.82 ± 2.39	9.08 ± 2.45
T_max_	h	7.33 ± 1.63	5.67 ± 2.34	6.33 ± 0.82
C_max_	μg/L	469.77 ± 194.97	686.48 ± 341.44	646.67 ± 350.99
V_z/F_	L/kg	35.93 ± 15.56	91.44 ± 65.54	126.53 ± 79.65
CL_z/F_	L/h/kg	2.77 ± 1.25	8.28 ± 4.17*	8.98 ± 3.83*

Note: Compared to control group, *P < 0.05. The results are expressed as mean ± SD.

### 3.3 Quercetin inhibited the metabolism of ziprasidone through a mixed inhibitory mechanism in both the RLM and HLM

**FIGURE 3:**
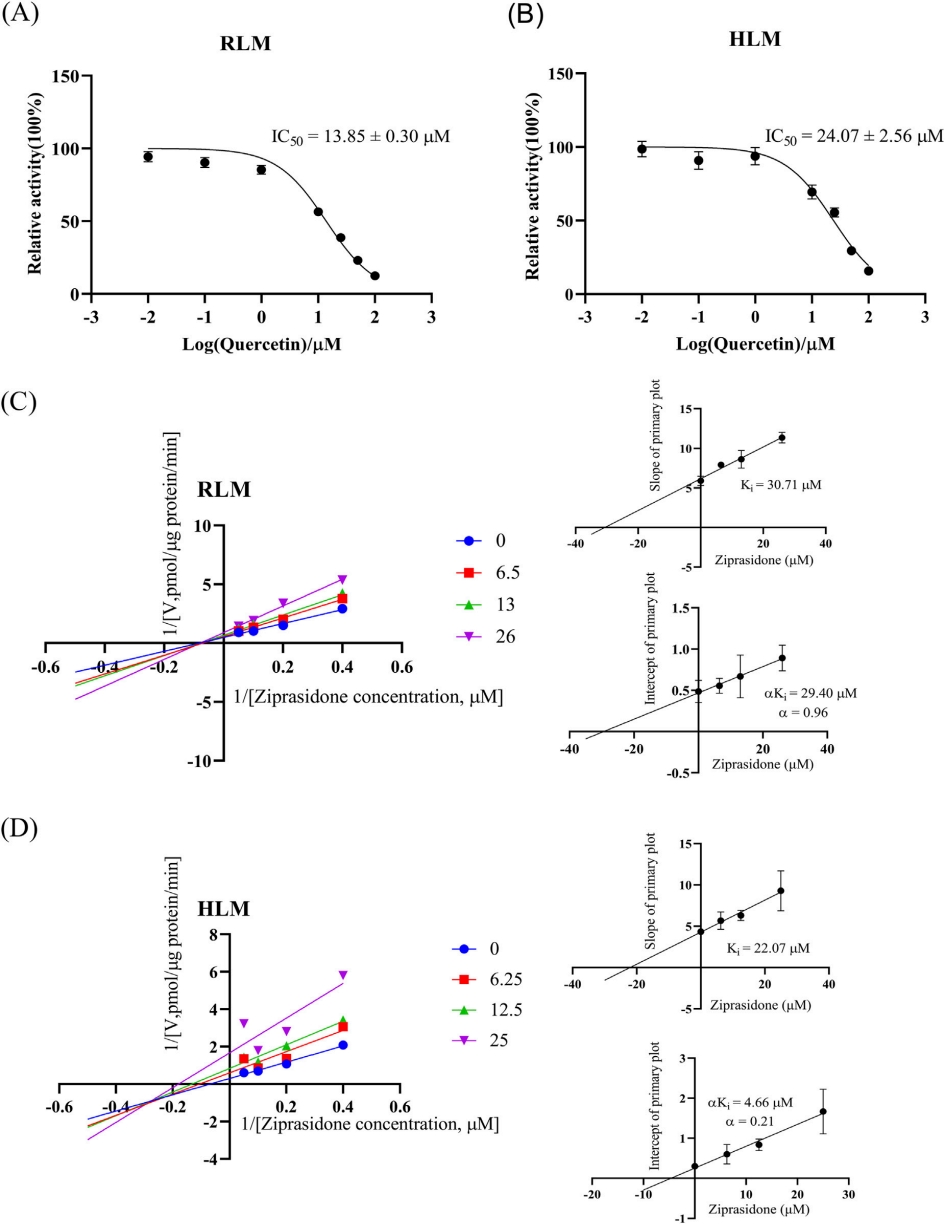
The inhibitory effect of quercetin on ziprasidone metabolism was characterized by a mixed mechanism in RLM and HLM. A range of quercetin concentrations (0, 0.01, 0.1, 1, 10, 25, 50 and 100 μM) with a fixed ziprasidone concentration (10 μM) was used to ascertain the half-maximal inhibitory concentration (IC_50_) values for ziprasidone metabolism by RLM **(A)** and HLM **(B)**. Data are presented as mean ± S.D., n = 3. Lineweaver-Burk plot and the secondary plot for K_i_ in inhibition of ziprasidone catalysis in RLM **(C)** and HLM **(D)**, n = 3.

Figure 3 and Table 3 show the IC_50_ curve and Line-Weaver-Burk plot of quercetin on ziprasidone metabolism in RLM and HLM incubation systems. Figure 3A, B showed that quercetin had a strong inhibitory effect on the metabolism of ziprasidone, with an IC_50_ value of 13.85 ± 0.30 μM in RLM microsomes and 24.07 ± 2.56 μM in HLM microsomes. As shown in Figures 3C,D Lineweaver-Burk, quercetin inhibited the metabolism of ziprasidone in both RLM and HLM through a mixed inhibitory mechanism. The inhibition constant (K_i_) of quercetin in RLM and HLM were 30.71 μM and 22.07 μM, respectively, and the corresponding αK_i_ values were 29.4 μM and 4.66 μM, respectively (α ≠ 1).

**TABLE 3:** The IC_50_ values and inhibitory effects of quercetin on ziprasidone metabolism in RLM, HLM and CYP3A4.1

Microsomes	IC_50_ values (μM)	Inhibition type	K_i_ (μM)	αK_i_ (μM)	α
RLM	13.85 ± 0.30	mixed inhibition	30.71	29.4	0.96
HLM	24.07 ± 2.56	mixed inhibition	22.07	4.66	0.21
CYP3A4.1	17.59 ± 1.01	mixed inhibition	15.18	10.06	0.66

### 3.4 Analysis of the activity of recombinant human CYP3A4 enzyme in ziprasidone metabolism

**FIGURE 4:**
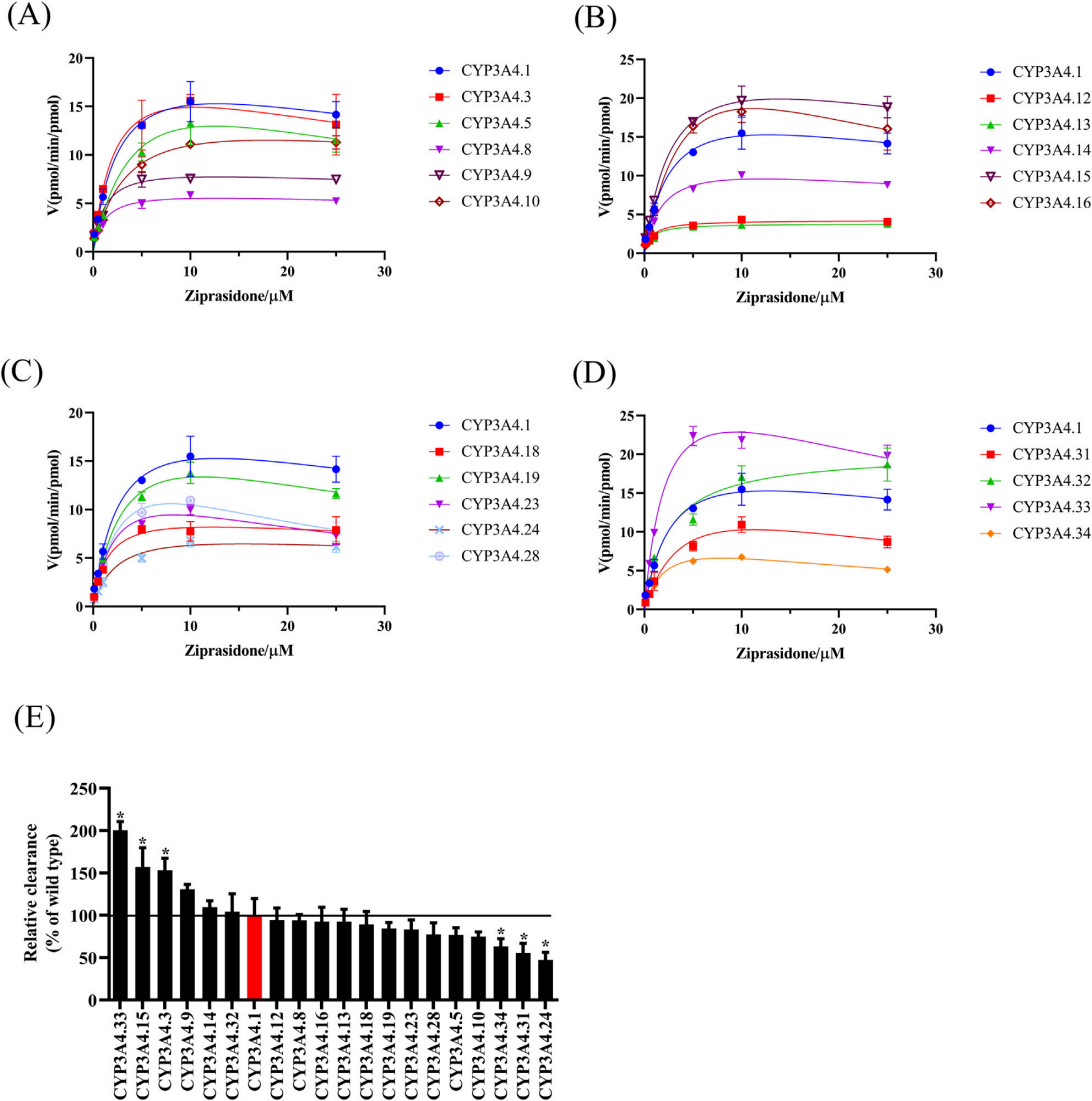
Michaelis-Menten plots for the CYP3A4-mediated metabolism of ziprasidone and the comparison of clearance rates among different CYP3A4 variants. **(A–D)** Michaelis-Menten curves of ziprasidone metabolism by wild-type CYP3A4 and other variants. V_max_ and K_m_ were obtained from Michaelis-Menten curves. CL = V_max_/K_m_. **(E)** The clearance rates of different variants were computed and graphically displayed in comparison to CYP3A4.1. Data are presented as mean ± SEM, n = 3. *P < 0.05.

Figure 4 shows the Michaelis-Menten curve of ziprasidone metabolism by wild-type CYP3A4.1 and other variants as well as the relative clearance histogram of all CYP3A4 variants examined in this experiment. The Michaelis kinetic parameters of ziprasidone in the CYP3A4 variant examined in this experiment are listed in Table 4. According to Table 4, there are two main changes in the maximum frequency response (V_max_): compared to CYP3A4.1, the V_max_ of CYP3A4. 8, 9, 10, 12, 13, 14, 18, 23, 24, 31, 32, 34 decreased from 13.84% to 72.98%. There was no significant change in other variants. Intrinsic clearance (CL) is considered the standard for assessing CYP3A4 enzyme activity. In this study, CL values were also divided into three categories: Compared with CYP3A4.1, CYP3A4.3, 15, 33 increased significantly, CL ranged from 153.21% to 242.83%; CYP3A4.24, 31, 34 were significantly reduced and the CL range was between 57% and 76.61%. There was no significant difference in other variants of CYP3A4.

**TABLE 4:** Kinetic parameters of ziprasidone catalysis in CYP3A4.

CYP	V_max_ (pmol/min/pmol)	K_m_ (pmol/μL)	CL (μL/min/pmol)	% of CYP3A4.1
CYP3A4.1	28.98 ± 4.56	5.04 ± 1.96	6.07 ± 1.19	100.00 ± 19.66
CYP3A4.3	22.59 ± 6.63	2.46 ± 0.85	9.30 ± 0.85*	153.26 ± 14.08*
CYP3A4.5	25.89 ± 11.32	5.80 ± 3.19	4.65 ± 0.52	76.73 ± 8.59
CYP3A4.8	6.55 ± 0.79*	1.15 ± 0.20	5.71 ± 0.42	94.14 ± 6.86
CYP3A4.9	9.15 ± 1.23*	1.16 ± 0.17	7.92 ± 0.36	130.65 ± 6.00
CYP3A4.10	16.26 ± 1.51*	3.59 ± 0.12	4.52 ± 0.35	74.60 ± 5.81
CYP3A4.12	4.43 ± 0.30*	0.79 ± 0.17	5.72 ± 0.87	94.29 ± 14.33
CYP3A4.13	4.01 ± 0.11*	0.73 ± 0.15	5.60 ± 0.90	92.30 ± 14.84
CYP3A4.14	12.70 ± 1.27*	1.93 ± 0.31	6.64 ± 0.48	109.41 ± 7.88
CYP3A4.15	29.84 ± 7.61	3.26 ± 1.31	9.53 ± 1.38*	157.07 ± 22.67*
CYP3A4.16	44.25 ± 19.83	7.07 ± 4.52	6.79 ± 1.26	92.32 ± 17.08
CYP3A4.18	11.05 ± 2.82*	1.78 ± 0.81	6.56 ± 1.12	89.27 ± 15.28
CYP3A4.19	22.26 ± 4.03	3.63 ± 0.85	6.19 ± 0.53	84.26 ± 7.20
CYP3A4.23	15.57 ± 3.00*	2.62 ± 0.82	6.11 ± 0.83	83.13 ± 11.26
CYP3A4.24	8.64 ± 1.20*	2.56 ± 0.63	3.46 ± 0.67*	47.09 ± 9.16*
CYP3A4.28	23.13 ± 10.24	4.36 ± 2.63	5.69 ± 1.01	77.40 ± 13.81
CYP3A4.31	19.11 ± 2.93*	4.80 ± 0.99	4.08 ± 0.83*	55.46 ± 11.30*
CYP3A4.32	21.15 ± 1.25*	2.87 ± 0.82	7.66 ± 1.55	104.25 ± 21.13
CYP3A4.33	34.60 ± 4.60	2.36 ± 0.42	14.74 ± 0.74*	200.60 ± 10.11*
CYP3A4.34	10.46 ± 1.22*	2.30 ± 0.58	4.65 ± 0.66*	63.31 ± 9.03*

Note: Compared to CYP3A4.1, *P < 0.05.

### 3.5 Variants of CYP3A4 can influence the inhibitory impact of quercetin

**FIGURE 5:**
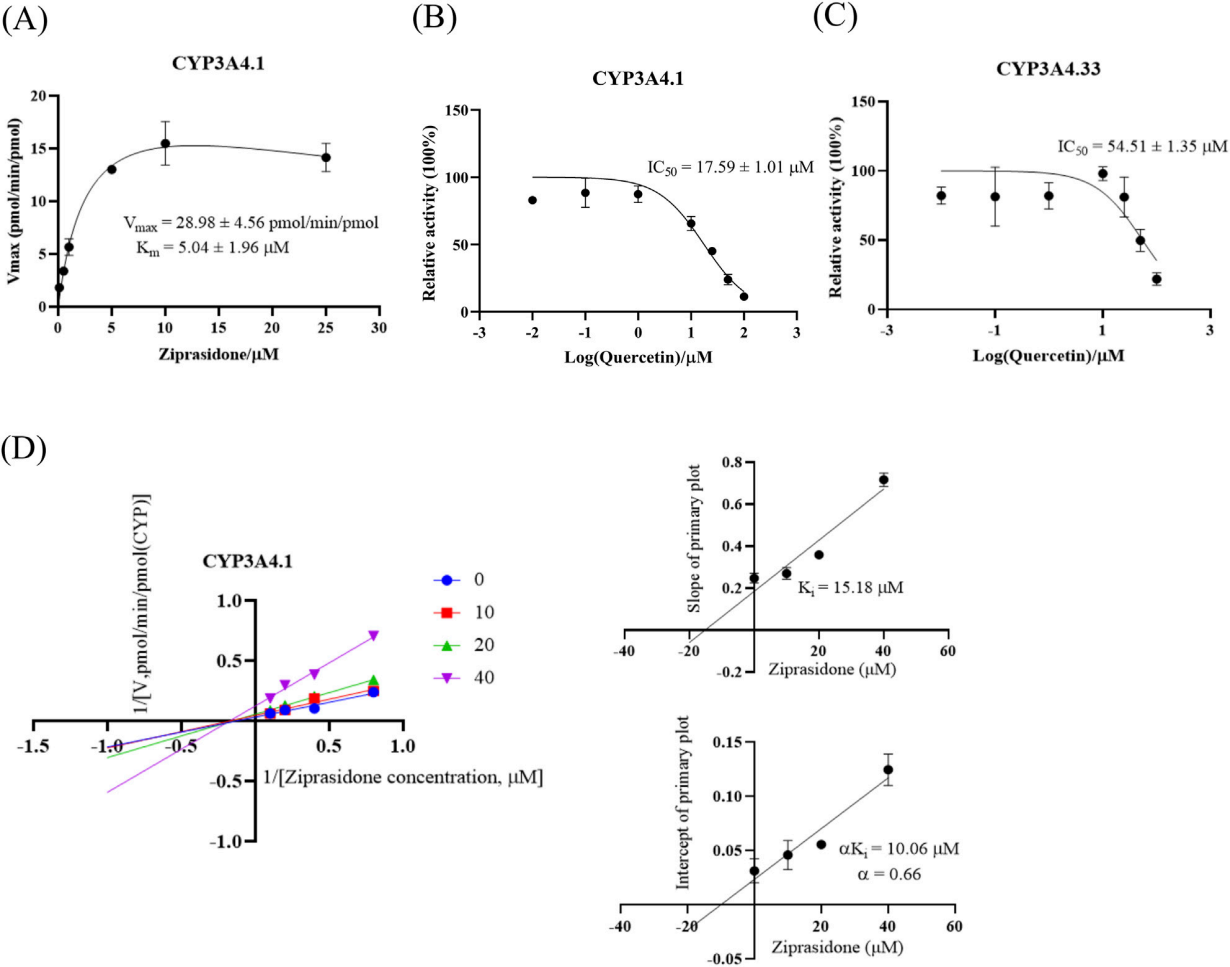
Related pharmacokinetic parameters of ziprasidone in the CYP3A4 enzyme system and the inhibitory mechanism of quercetin. **(A)** Michaelis-Menten curves of ziprasidone metabolism in CYP3A4.1. n = 3. A series of quercetin concentrations (0, 0.01, 0.1, 1, 10, 25, 50, 100 μM) were applied to ascertain the half-maximal inhibitory concentration (IC_50_) of ziprasidone metabolism by CYP3A4.1. **(B)** and CYP3A4.33 **(C)**. Data are presented as mean ± S.D., n = 3. **(D)** Lineweaver-Burk plot and the secondary plot for K_i_ in inhibition of ziprasidone catalysis in CYP3A4.1, n = 3.

The CYP3A4 genetic polymorphism dictates the pharmacokinetic profile of ziprasidone and may potentially influence the drug’s inhibitory effectiveness. We chose CYP3A4.33, which has a significantly higher clearance rate than CYP3A4.1, for further study. The results are shown in Figure 5 and Table 3. As shown in Figure 5A, in the CYP3A4.1 incubation system, the V_max_ of ziprasidone was 28.98 ± 4.56 pmol/min/pmol and the K_m_ value was 5.04 ± 1.96 μM. Figures 5B, C show that quercetin still has a strong inhibitory effect on the metabolism of ziprasidone in CYP3A4.1 microsomes with an IC_50_ value of 17.59 ± 1.01 μM. In the CYP3A4.33 incubation system, the IC_50_ value of quercetin was 54.51 ± 1.35 μM. As shown in Figure 5D Lineweaver-Burk, quercetin exerted a mixed-type inhibitory mechanism on the metabolism of ziprasidone in the CYP3A4.1 incubation system. The inhibition constant K_i_ was 15.18 μM and the corresponding αK_i_ value was 10.06 μM (α ≠ 1).

**FIGURE 6:**
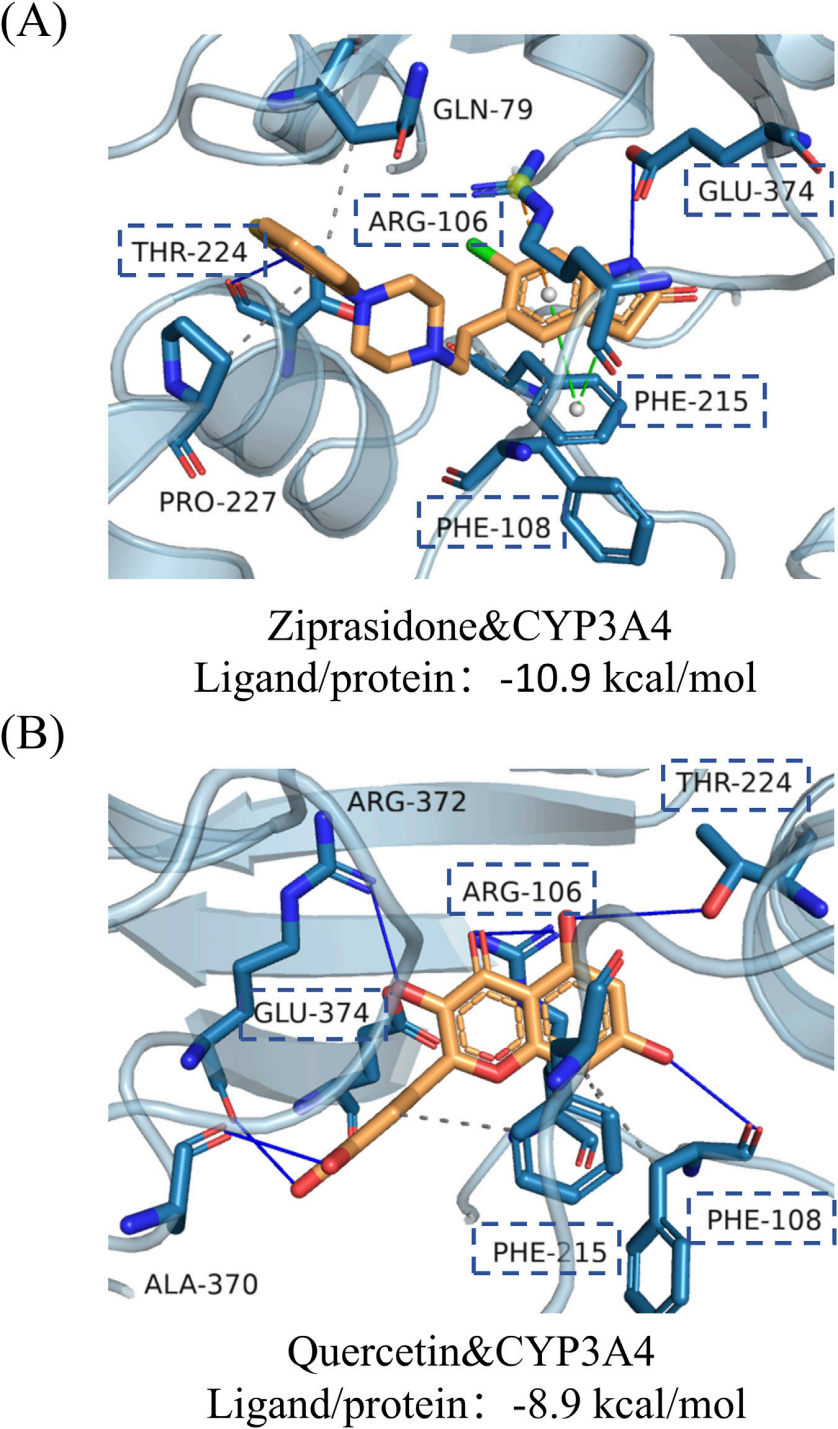
Molecular docking results of drugs with CYP3A4 enzyme. Using key targets as receptors and their corresponding effective components as ligands, molecular docking was performed using Vina in PyrX software, the binding energy was calculated, and the results file was output. Finally, the results are visualized using the PyMol software. Molecular docking of ziprasidone **(A)** and quercetin **(B)** to the enzyme CYP3A4.

We conducted further research into the molecular underpinnings of this inhibitory effect. As shown in Figure 6A, it is based on the molecular docking of the CYP3A4 enzyme 1tqn protein chain and the coenzyme binding mode. 1tqn interacts with GLN79, PHE108, PHE215, and PRO227 to form permanent hydrophobic interactions, interacts with THR224 and GLU374 to form hydrogen bonds, interacts with PHE215 to form π-stacking interactions, and interacts with ARG106 to form π-cation to form interactions. The docking binding energy of 1tqn is −10.9 kcal/mol. Similarly, Figure 6B shows the binding mode of 1tqn and quercetin. 1tqn interacts with PHE108 and PHE215 of quercetin target proteins to form hydrophobic interactions and interacts with ARG106, PHE108, THR224, ALA370, ARG372, GLU374 to form hydrogen bonding interactions. The docking results showed that the binding energy of 1tqn and quercetin was −8.9 kcal/mol. Quercetin may inhibit the metabolism of ziprasidone by CYP3A4 enzyme by competing with PHE108, PHE215, THR224, GLU374 and ARG106.

## 4 Discussion

Ziprasidone is an atypical antipsychotic approved by the FDA for the treatment of schizophrenia (Harvey and Bowie, 2005). Schizophrenia is a mental illness and one of the top ten causes of long-term disability worldwide. Schizophrenic patients make up about 0.3%–1% of the total population (Mueser and McGurk, 2004). At the same time, patients with schizophrenia have a significantly increased risk of developing cardiovascular diseases, metabolic syndrome, obesity, hypertension, and hyperlipidemia (Bueno-Antequera and Munguía-Izquierdo, 2020). Therefore, compared to the general population, the life expectancy of patients with schizophrenia is almost 20% shorter (Hennekens, 2007; Nielsen et al., 2021). It is precisely due to the presence of several complications that psychiatric patients often take multiple medications concurrently, which can easily lead to interactions between the drugs and consequently cause fluctuations in the plasma concentrations of the medications.

Herein, we evaluated potential drug-drug interactions between ziprasidone and cardiovascular/natural compounds, identifying resveratrol and quercetin as potent inhibitors of ziprasidone metabolism in RLM. Translating clinically relevant doses to rats, we found that quercetin coadministration significantly increased ziprasidone’s AUC and C_max_, while unexpectedly, ziprasidone sulfoxide metabolite levels showed a non-significant elevation—a paradoxical observation given that CYP3A4 inhibition typically reduces metabolite formation. We propose this may result from insufficient systemic quercetin concentrations to achieve full enzyme inhibition *in vivo* and/or compensatory metabolic pathway activation, as ziprasidone undergoes multiple elimination routes. Pharmacokinetic analysis revealed an unchanged t_1/2_, suggesting primary intestinal (rather than hepatic) CYP3A4 interaction, consistent with first-pass effects (Grenier et al., 2006). while increased V_z/F_ and CL_z/F_ likely reflect reduced bioavailability rather than true distribution/clearance changes (Sodhi et al., 2021). As resveratrol exhibited negligible *in vivo* inhibition, we focused on quercetin, concluding that its interaction with ziprasidone involves complex interplay between metabolic inhibition, compensatory pathways, and bioavailability alterations, warranting further mechanistic investigation.

Resveratrol and quercetin are flavonoids - natural bioactive compounds widely present in common foods and herbal medicines, including various fruits and vegetables (Cushnie and Lamb, 2005). They possess anti-inflammatory, anticancer, lipid-lowering, and hypoglycemic effects, etc. (Rakha et al., 2022). Currently, people tend to use natural products more than synthetic drugs to enhance their health conditions. Flavonoids are less toxic and consume a lot in our daily life (Li et al., 2018). At the same time, dietary supplements are becoming increasingly popular as part of alternative natural therapies to combat obesity among psychiatric patients, and flavonoids are often used in high doses as dietary supplements by patients (Oga et al., 2016; Wu et al., 2016; Bhutani et al., 2020). The components of dietary supplements may be substrates, inducers and/or inhibitors of drug metabolism enzymes and/or transporters that are responsible for the pharmacokinetics of antipsychotics and thus influence the metabolism of ziprasidone (Gurley, 2012; Cho and Yoon, 2015; Choi and Song, 2019). Because flavonoid compounds are often used by patients as dietary supplements, food additives, and medications, the potential for drug interactions with these substances deserves increasing attention.

To further characterize the interaction between quercetin and ziprasidone, we conducted *in vitro* experiments using rat (RLM) and human (HLM) liver microsomes to assess quercetin’s inhibitory potential and elucidate its mechanism of action. The IC_50_ values demonstrated that quercetin significantly inhibits ziprasidone metabolism in both RLM and HLM systems, consistent with our *in vivo* observations. Kinetic analysis revealed that quercetin acts as a mixed-mechanism inhibitor of ziprasidone metabolism in both species. We propose that this inhibition primarily arises from quercetin’s ability to suppress cytochrome P450 (CYP450) enzymes, particularly CYP3A, thereby altering ziprasidone’s metabolic clearance and systemic exposure. Notably, our data revealed interspecies differences in ziprasidone metabolism, which we hypothesize may stem from variations in CYP3A activity between rats and humans.

CYP3A4, the primary enzyme responsible for ziprasidone metabolism (yielding ziprasidone sulfoxide as the major metabolite), exhibits significant genetic polymorphism that leads to substantial interindividual variability in enzymatic activity. These functional differences, stemming from genetic variations in the CYP3A4 sequence, result in distinct metabolic phenotypes, which contribute to the observed spectrum of therapeutic outcomes ranging from subtherapeutic effects to adverse drug reactions. Despite ziprasidone’s widespread clinical use and documented safety concerns, the influence of CYP3A4 polymorphisms on its metabolism remains understudied. To address this gap, we systematically evaluated 19 CYP3A4 variants against wild-type CYP3A4.1, identifying three hyperactive variants (CYP3A4.3, .15, and .33) that demonstrated enhanced catalytic activity, suggesting carriers may require dose escalation or increased administration frequency while maintaining therapeutic drug monitoring to mitigate toxicity risks. Conversely, CYP3A4.24, .31, and .34 exhibited significantly reduced V_max_ values and diminished catalytic capacity. Patients carrying these variants may exhibit reduced metabolism of ziprasidone, potentially leading to elevated drug concentrations in the body following administration. Consequently, dose reduction or extension of the dosing interval may be considered. If necessary, therapeutic drug monitoring could be performed to minimize the risk of adverse effects and potential drug interactions. While these findings require *in vivo* validation, our *in vitro* characterization of CYP3A4 variant-specific metabolic profiles provides crucial preliminary evidence for personalized ziprasidone dosing strategies in clinical practice.

Furthermore, we investigated how CYP3A4 genetic variations influence drug-drug interactions. Our findings demonstrate that quercetin exhibits stronger inhibition of ziprasidone metabolism mediated by wild-type CYP3A4.1 compared to the hyperactive variant CYP3A4.33. This differential effect may stem from CYP3A4.33's enhanced catalytic efficiency toward ziprasidone, which accelerates substrate binding and reduces quercetin’s occupancy at competitive sites, thereby diminishing its inhibitory potency. These observations suggest that CYP3A4 polymorphisms can significantly modulate the magnitude of metabolic drug interactions. To elucidate the inhibitory mechanism, we focused on the CYP3A4.1 incubation system and determined that quercetin acts as a mixed-type inhibitor of ziprasidone metabolism. Molecular docking simulations revealed that quercetin competitively binds to key residues (PHE108, PHE215, THR224, GLU374, and ARG106) within the CYP3A4 active site, directly interfering with ziprasidone-enzyme interactions. While this study provides mechanistic insights, we acknowledge that our analysis of a single variant (CYP3A4.33) represents only a fraction of clinically relevant polymorphisms. Further investigations incorporating additional genotypes are warranted to establish comprehensive evidence for personalized drug interaction predictions.

In summary, this study elucidates the dual impact of CYP3A4 genetic polymorphisms and quercetin-mediated drug interactions on ziprasidone metabolism. Our findings demonstrate that quercetin significantly inhibits ziprasidone metabolism through mixed-type inhibition of CYP3A4, which may lead to clinically relevant pharmacokinetic alterations. These results suggest that co-administration of quercetin with ziprasidone in psychiatric patients could potentially elevate drug exposure, increasing the risk of adverse effects. Consequently, clinicians should exercise caution when prescribing these compounds concomitantly, with consideration given to potential dose reduction of ziprasidone under therapeutic drug monitoring. Furthermore, our characterization of CYP3A4 variants reveals substantial interindividual variability in ziprasidone metabolic capacity, highlighting the importance of pharmacogenetic profiling for personalized dosing strategies. While these findings provide valuable mechanistic insights into ziprasidone pharmacokinetics, further clinical validation is warranted to establish definitive genotype-phenotype correlations. Collectively, this work establishes a foundation for optimizing ziprasidone therapy through both drug interaction management and precision medicine approaches.

## Data Availability

The datasets presented in this study can be found in online repositories. The names of the repository/repositories and accession number(s) can be found in the article/Supplementary Material.
