# Supplementary material for: Impact of CYP3A4 functional variability on ziprasidone metabolism
Source: Front Pharmacol. 2025 Apr 29;16:1585040. doi: 10.3389/fphar.2025.1585040 (PMC12069442; doi:10.3389/fphar.2025.1585040)
Supplement: Supplementary file 1 [file DataSheet1.docx]

**Impact of CYP3A4 Functional Variability on Ziprasidone Metabolism**

**Running head: Variation in ziprasidone metabolism**

Qi Zhou^1^^,2,#^, Yameng Wu^1,#^, Zhize Ye^3^, Zheyan Zhang^2^, Kai Zheng^1^, Jianchang Qian^2,*^, Zhongxiang Xiao^1,*^, Yang Lu^1,*^

^1^ Affiliated Yueqing Hospital, Wenzhou Medical University, Wenzhou, Zhejiang, China

^2^ Institute of Molecular Toxicology and Pharmacology, School of Pharmaceutical Sciences, Wenzhou Medical University, Wenzhou, Zhejiang, China

^3^ Department of Pharmacy, Shaoxing People’s Hospital, Shaoxing, China

# These authors contribute equally to the work.

*Corresponding authors:

Jianchang Qian, School of Pharmaceutical Sciences, Wenzhou Medical University, Wenzhou, Zhejiang, China. Telephone: 15068233876. E-mail: qianjc@wmu.edu.cn.

Zhongxiang Xiao, Affiliated Yueqing Hospital, Wenzhou Medical University, E-mail: xiangzi198155@163.com

Yang Lu, Affiliated Yueqing Hospital, Wenzhou Medical University, E-mail: 190465626@qq.com

(A) (B) (C)





**Fig. S1** Representative chromatograms of ziprasidone, ziprasidone sulfoxide and internal standard (IS). (A) Chromatogram of a blank plasma sample. (B) Chromatogram of a blank plasma sample supplemented with ziprasidone, ziprasidone sulfoxide, and midazolam. (C) Chromatogram of a rat plasma sample.

Table S1. Inhibition rate of inhibitors on the metabolism of ziprasidone in RLM.

| **CAS** | **Inhibitor** | **Relative activity (%)** |
| --- | --- | --- |
| 529-44-2 | Myricetin | 2.64 ± 1.71 |
| 117-39-5 | Quercetin | 17.67 ± 2.93 |
| 501-36-0 | Resveratrol | 18.28 ± 12.02 |
| 141625-93-6 | Dronedarone Hydrochloride | 23.67 ± 11.05 |
| 88150-42-9 | Amlodipine | 24.44 ± 2.51 |
| 518-82-1 | Emodin | 25.64 ± 3.15 |
| 86189-69-7 | Felodipine | 28.36 ± 1.67 |
| 72956-09-3 | Carvedilol | 29.89 ± 3.28 |
| 520-36-5 | Apigenin | 31.41 ± 2.56 |
| 124750-99-8 | Losartan potassium | 35.58 ± 50.31 |
| 480-40-0 | Chrysin | 36.09 ± 19.09 |
| 184475-35-2 | Gefitinib | 36.20 ± 0.61 |
| 274693-27-5 | Ticagrelor | 36.84 ± 20.49 |
| 1446502-11-9 | Enasidenib | 40.44 ± 1.29 |
| 137862-53-4 | Valsartan | 44.50 ± 62.94 |
| 22888-70-6 | Silibinin | 45.13 ± 1.52 |
| 66085-59-4 | Nimodipine | 49.68 ± 0.53 |
| 139481-59-7 | Candesartan | 54.29 ± 3.95 |
| 73963-72-1 | Cilostazol | 55.05 ± 0.99 |
| 152520-56-4 | Nebivolol Hydrochloride | 63.48 ± 3.34 |
| 144701-48-4 | Telmisartan | 64.19 ± 12.39 |
| 61281-38-7 | Schisandrin A | 69.51 ± 11.79 |
| 138402-11-6 | Irbesartan | 72.41 ± 4.72 |
| 114798-26-4 | Losartan | 74.09 ± 5.29 |
| 4205-91-8 | Clonidine hydrochloride | 74.70 ± 0.47 |
| 632-85-9 | Wogonin | 80.85 ± 12.40 |
| 56-54-2 | Quinidine | 83.40 ± 4.78 |
| 17086-76-9 | Cyasterone | 83.75 ± 6.16 |
| 38748-32-2 | Triptolide | 87.47 ± 5.70 |
| 21967-41-9 | Baicalin | 87.83 ± 13.63 |
| 327-97-9 | Chlorogenic acid | 91.52 ± 17.97 |
| 51059-44-0 | Wogonoside | 92.55 ± 1.09 |
| 147403-03-0 | Azilsartan | 92.59 ± 5.86 |
| 21967-41-9 | Baicalin | 92.59 ± 8.76 |
| 137-58-6 | Lidocaine | 93.56 ± 10.94 |
| 122-48-5 | Vanillylacetone | 93.56 ± 3.00 |
| 491-67-8 | Baicalein | 97.20 ± 18.86 |
| 519-02-8 | Matrine | 98.65 ± 1.77 |
| 366789-02-8 | Rivaroxaban | 102.37 ± 4.89 |
| 98717-15-8 | Ropivacaine hydrochloride | 102.42 ± 0.84 |
| 64849-39-4 | Rubusoside | 103.75 ± 2.05 |
| 14252-80-3 | Bupivacaine hydrochloride | 104.14 ± 92.28 |
| 20575-57-9 | Calycosin | 104.22 ± 12.51 |
| 84775-42-8 | Anise oil | 105.07 ± 11.61 |
| 83207-58-3 | Astragaloside A | 107.37 ± 18.93 |
| 552-41-0 | Paeonol | 108.77 ± 3.51 |
| 614-39-1 | Procainamide hydrochloride | 113.37 ± 13.44 |
| 6199-67-3 | Cucurbitacin B | 117.16 ± 18.74 |
| 486-66-8 | Daidzein | 121.70 ± 5.16 |
| 120202-66-6 | Clopidogrel hydrogen sulfate | 179.48 ± 15.80 |

Table S2. The information of CYP3A4 alleles and mutation sites.

| **Allele** | **Protein** | **Nucleotide change (NM_017460.6)** | **Impact (NP_059488.2)** | **rsIDs** |
| --- | --- | --- | --- | --- |
| CYP3A4*1 | CYP3A4.1 | None | None | None |
| CYP3A4*3 | CYP3A4.3 | c. 1334T>C | p. M445T | rs4986910 |
| CYP3A4*5 | CYP3A4.5 | c. 653C>G | p. P218R | rs55901263 |
| CYP3A4*8 | CYP3A4.8 | c. 389G>A | p. R130Q | rs72552799 |
| CYP3A4*9 | CYP3A4.9 | [c. 508G>A](https://www.ncbi.nlm.nih.gov/snp/rs72552798) | p. V170I | rs72552798 |
| CYP3A4*10 | CYP3A4.10 | [c. 520G>C](https://www.ncbi.nlm.nih.gov/snp/rs4986908) | p. D174H | rs4986908 |
| CYP3A4*12 | CYP3A4.12 | c. 1117C>T | p. L373F | rs12721629 |
| CYP3A4*13 | CYP3A4.13 | [c. 1247C>T](https://www.ncbi.nlm.nih.gov/snp/rs4986909) | p. P416L | rs4986909 |
| CYP3A4*14 | CYP3A4.14 | c. 44T>C | p. L15P | rs12721634 |
| CYP3A4*15 | CYP3A4.15 | [c. 485G>A](https://www.ncbi.nlm.nih.gov/snp/rs4986907) | p. R162Q | rs4986907 |
| CYP3A4*16 | CYP3A4.16 | c. 554C>G | p. T185S | rs12721627 |
| CYP3A4*18 | CYP3A4.18 | [c. 878T>C](https://www.ncbi.nlm.nih.gov/snp/rs28371759) | p. L293P | rs28371759 |
| CYP3A4*19 | CYP3A4.19 | [c. 1399C>T](https://www.ncbi.nlm.nih.gov/snp/rs4986913) | p. P467S | rs4986913 |
| CYP3A4*23 | CYP3A4.23 | c. 484C>T | p. R162W | rs57409622 |
| CYP3A4*24 | CYP3A4.24 | c. 600A>T | p. Q200H | rs113667357 |
| CYP3A4*28 | CYP3A4.28 | c. 64C>G | p. L22V | rs570051168 |
| CYP3A4*31 | CYP3A4.31 | c. 972C>A | p. H324Q | rs1303250043 |
| CYP3A4*32 | CYP3A4.32 | c. 1004T>C | p. I335T | rs368296206 |
| CYP3A4*33 | CYP3A4.33 | c. 1108G>T | p. A370S | rs756833413 |
| CYP3A4*34 | CYP3A4.34 | c. 1279A>G | p. I427V | rs774109750 |
